# Efficacy and Predictability of Maxillary and Mandibular Dental Arch Expansion with Clear Aligners in Prepuberal Subjects: A Digital Retrospective Analysis

**DOI:** 10.3390/healthcare13131508

**Published:** 2025-06-24

**Authors:** Silvia Caruso, Alessandro Nota, Chiara Tonelli, Sandra Khong Tai, Gianluca Baldini, Fabiana Fiasca, Sara Caruso, Antonella Mattei

**Affiliations:** 1 Department of Life, Health and Environmental Sciences, University of L’Aquila, 67100 L’Aquila, Italy; silvia.caruso@univaq.it (S.C.); gianluca.baldini@student.univaq.it (G.B.); fabiana.fiasca@alice.it (F.F.); sara.caruso@graduate.univaq.it (S.C.); antonella.mattei@univaq.it (A.M.); 2 Dental School, IRCCS San Raffaele Hospital, Vita-Salute San Raffaele University, 20132 Milano, Italy; nota.alessandro@hsr.it; 3 Department of Orthodontics, University of British Columbia, Vancouver, BC V6T 1Z4, Canada; sandra@drsandratai.com

**Keywords:** dental expansion, mixed dentition, aligners

## Abstract

**Background/Objectives**: Previous studies on clear aligner therapy (CAT) in mixed dentition primarily focused on the predictability of maxillary arch expansion. However, limited evidence is available regarding mandibular arch changes, particularly in relation to inter-arch coordination. This study aims to evaluate the effectiveness and predictability of dental expansion in both the upper and lower arches using Invisalign First^®^ aligners. **Methods**: A retrospective analysis was conducted with 15 participants. Dental expansions were assessed before and after treatment using iTero intraoral scans processed with 3D analysis software. Measurements were compared to the predicted movements planned in ClinCheck^®^. Data normality was verified (Shapiro–Wilk test), descriptive statistics were calculated, and paired t-tests were performed to compare clinical and predicted expansions, with significance set at 0.05. **Results**: Clear aligners achieved effective dento-alveolar expansion in both arches. Predictability was higher at the cusp level than at the gingival level, indicating a tendency toward tipping movements rather than bodily expansion. The study also highlighted mandibular expansion outcomes and gingival-level discrepancies, providing new insights compared to the previous literature. Minor differences between predicted and achieved movements were observed, partly attributable to natural growth and deciduous tooth exfoliation. **Conclusions**: Clear aligners are effective in achieving maxillary and mandibular arch expansion in mixed dentition, with good predictability at the coronal level. Overengineering buccal root torque may help promote bodily expansion and reduce cuspal–gingival discrepancies. Further studies with larger sample sizes are needed to optimize treatment planning and predictability.

## 1. Introduction

Dental arch expansion has long been a debated topic in the literature, with different orthodontic techniques and appliances applied to both adults and growing patients. This issue is particularly relevant in non-extraction treatments, where managing moderate crowding and narrow arches requires increasing the arch perimeter through transverse expansion and/or incisor proclination [1,2].

Traditionally, transverse maxillary deficiency has been addressed using both removable and fixed appliances, such as the Schwarz plate with expansion screws, transpalatal arches (TPA), and rapid palatal expanders (e.g., Hyrax). These methods are especially effective in children due to the greater plasticity of craniofacial sutures during growth. In adult patients, however, skeletal expansion becomes more challenging and may require surgical assistance, such as surgically assisted rapid palatal expansion (SARPE), to achieve stable outcomes.

Over the past two decades, clear aligner therapy (CAT) has become increasingly popular, especially among adult patients seeking aesthetic and comfortable alternatives to conventional fixed appliances [3,4,5,6]. Unlike most orthodontic techniques, CAT is based on a digital workflow: after acquiring a 3D model of the dental arches, tooth movements are virtually planned using dedicated software, enabling precise control over direction, magnitude, and timing. Nevertheless, not all planned movements are fully achieved clinically, even when appropriate biomechanics (e.g., attachments) are applied. Systematic reviews have shown that the predictability of movement can vary depending on the type of movement [7].

Several studies have analyzed the biomechanics of arch expansion using conventional appliances [8,9], but only two studies have investigated dentoalveolar expansion using clear aligners, and both were conducted on the upper arches of adult subjects [1,10]. These studies reported that CAT can produce arch expansion mainly through buccal tipping, particularly of the posterior maxillary teeth, but they also highlighted limited control over canine movement.

The Invisalign First^®^ protocol was developed specifically for interceptive treatment in growing patients (ages 6–10) and allows for arch development, space management, and alignment in primary and early mixed dentition. Its programming includes both sequential and simultaneous expansion of the upper arch as a guide arch.

The literature review by Caruso et al. [11] reminds us that the focus is on considering software as a system of forces designed to move teeth and not as a predictor of tooth movement.

As CAT allows for better oral hygiene and less caries risk during the orthodontic treatment, comfort, versatility, and greater patient acceptance, its use was recently introduced in the presence of crowding in growing subjects with mixed dentition, where dental crowding is commonly observed [12,13,14].

Thanks to improved comfort, better hygiene, and higher patient acceptance, clear aligner therapy has recently been extended to pediatric populations. In particular, it is now used in patients with mixed dentition to treat dental crowding and perform dentoalveolar expansion, especially in the lower arch, where skeletal expansion is not feasible. It should be noted that in prepubertal patients, anatomical and metabolic factors (e.g., dental morphology, bone remodeling rate) may influence the predictability of expansion.

Therefore, the aim of this study is to investigate the efficacy and predictability of maxillary and mandibular arch expansion in prepubertal subjects with mixed dentition using a simultaneous digital programming protocol for both arches.

## 2. Materials and Methods

A sample of 15 subjects were retrospectively included in the study, including 8 females and 7 males, with an average age of 9 ± 2.1 years. The protocol was in accordance with the Declaration of Helsinki, and it was ethically approved by the University of L’Aquila (Document DR 206/2013). Informed consent was obtained from the parents of each patient.

Each of them underwent orthodontic dento-alveolar expansion treatment with clear aligner therapy (CAT) (Invisalign First^®^, Align Technology Inc., Tempe, AZ, USA). The average duration of treatment was 8 months, with an average number of 33 aligners with a 7-day change protocol. The aligners were worn for 22 h per day.

The following inclusion criteria were applied: Caucasian ethnicity, age between 6 and 12 years, mixed dentition, fully erupted maxillary and mandibular first molars, posterior transverse interarch discrepancy up to a 6 mm (calculated by making the difference between the maxillary intermolar distance from the central fossa of one maxillary molar to the other and the mandibular intermolar distance between the cusps of the right and left mandibular molars) arch expansion planned with Invisalign^®^ First protocol, and good compliance with aligners.

Subjects with the following characteristics were excluded from the sample: previous orthodontic treatment, presence of craniofacial anomalies or syndromes, extraction cases, dental agenesis, supernumerary teeth, advanced or multiple cavities, periodontal illness, and a need for myofunctional treatment and muscle re-education.

### 2.1. Treatment Protocol

The patients underwent non-extraction orthodontic treatment with the Invisalign First System^®^ without using any auxiliary device other than Invisalign^®^ attachments and without resorting to interproximal reduction (IPR) of the enamel.

For the maxillary arch, each patient’s ClinCheck^®^ software (Align Technology Inc., San Jose, CA, USA; version 6.0) was planned with the same standardized expansion protocol “molars move first”, followed by simultaneous expansion of all posterior primary teeth and canines. The amount of arch expansion was 0.25 mm per stage.

Furthermore, for the maxillary first molars, a simultaneous derotation following Rickett’s line [15] and 2 degrees of extra buccal root torsion for each expansion phase were applied. An overcorrection of the superior transverse dimension was never prescribed, as a cusp–fossa relationship was digitally planned in all subjects.

The expansion in the lower arch, necessary to obtain transverse coordination between the shape of the upper and lower arch [16,17], was performed by a default protocol with “molar first” expansion with a programmed expansion of 4–6 mm per hemiarch.

All patients were instructed to wear their aligners full-time except during meals and when brushing teeth. The first aligner was worn for 10 days, while the subsequent ones were changed every 7 days, and the subjects were monitored by the orthodontist every 4 aligners to check the correct fit of the aligner, and any missing attachments were replaced.

Optimized expansion support attachments and optimized retentive attachments were automatically positioned by the software, adapting them to the size of the buccal surface of the posterior teeth.

### 2.2. Measurement Protocol

Pre-treatment (T0) and post-treatment (T1) digital dental models, obtained from an iTero^®^ intraoral scanner (Align Technology Inc., Tempe, AZ, USA), and digital.stl files were loaded into three-dimensional digital software (GOM Inspect 2019, Braunschweig, Germany).

Measurements are obtained from the center of the cusp tips between one tooth and the other.

**Figure 1:**
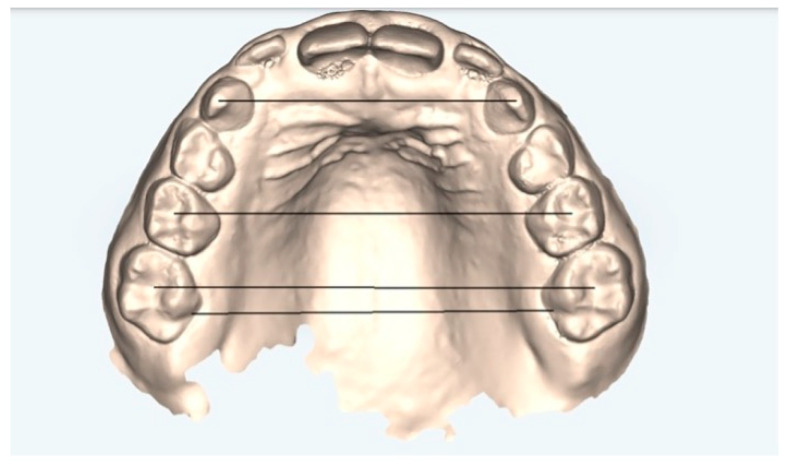
Upper arch measurements: digital dental model obtained by an iTero^®^ intraoral scanner (Align Technology Inc., Tempe, AZ, USA).

**Figure 2:**
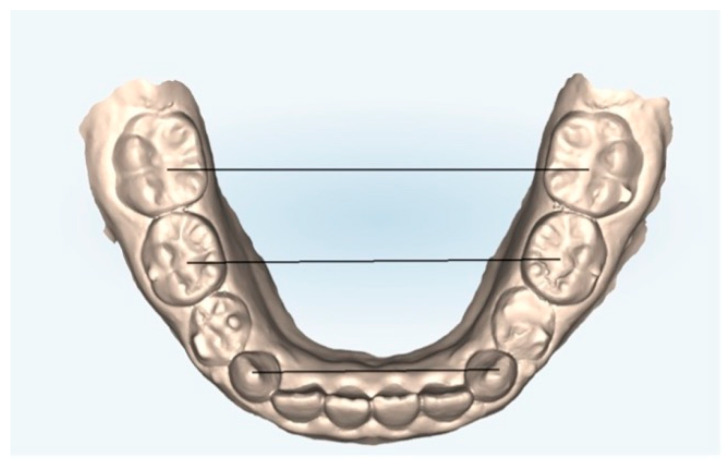
Lower arch measurements: digital dental model obtained by an iTero^®^ intraoral scanner (Align Technology Inc., Tempe, AZ, USA).

The following measurements (Figure 1 and Figure 2) were performed on both the arches models at T0 and T1 and on the models obtained from the ClinCheck^®^ final position of each treatment plan (T1C):

- Canine cuspid width (CCW): linear distance between the tips of the cusps of the upper or lower canines;
- Canine gingival width (CGW): linear distance from the center of a palatal surface in contact with the gingival margin of one canine, upper or lower, to the other;
- First primary molar cuspid width (1PMWC): linear distance between the tips of the buccal cusps of the upper or lower first premolars;
- First primary molar gingival width (1PMWG): linear distance between the center of the palatal surface in contact with the gingival margin of the upper or lower first premolars;
- Second primary molar cuspid width (2PMWC): linear distance between the tips of the buccal cusps of the upper or lower second premolars;
- Second primary molar gingival width (2PMWG): linear distance between the center of the palatal surface in contact with the gingival margin of the upper or lower second premolars;
- First permanent molar cuspid width (MWC): linear distance between the tips of the buccal cusps of the upper or lower first permanent molars;
- First permanent molar gingival width (MWG): linear distance between the center of the palatal surface in contact with the gingival margin of the upper or lower first permanent molars.

The measurements obtained and their variations were statistically compared to observe the differences between the final position of the post-treatment models (T1) and the final ClinCheck^®^ position (T1C) and to evaluate the predictability of the ClinCheck^®^ treatment plan.

### 2.3. Statistical Analysis

After testing for the existence of a normal distribution of the datasets (Shapiro–Wilk test), descriptive statistics were performed using the mean and standard deviation (SD). Furthermore, to determine the consistency between the measurements performed by the 3DModel and the ClinCheck^®^ position, the clinical and predicted measurement variations (ΔClinical = T1 − T0; ΔPredicted = T1C − T0) were calculated, and pairwise comparisons were performed using a paired t-test, and a significance threshold of 0.05 was applied.

Intraclass correlation coefficients (ICCs) with a two-way random effects model were used to analyze the reliability of the clinical and virtual measurements of expansion motion, according to Terry [18].

Based on the 95% confidence interval of the estimated ICC, reliability was considered low for values less than 0.5, moderate for values between 0.5 and 0.75, good between 0.75 and 0.9, and excellent for values above 0.90 [18].

Statistical analysis was performed using the STATA statistical package (Stata Statistical Software: Release 17—Stata Corp LP, College Station, TX, USA).

## 3. Results

The results for the maxillary and mandibular arches are presented separately. Only the main indicators (CCW, 1PMWC, MWC, and MWG) are included in the main text for clarity, while all other variables and full data tables are available in the Supplementary Materials.

### 3.1. Upper Arch

Pre-treatment and post-treatment clinical measurements were compared, and the difference between T1 and T0 is reported under the clinical delta column. On all measured levels, a satisfactory expansion rate was achieved.

Pre-treatment measurements were then compared to the ClinCheck^®^ predicted expansion value, and the difference was recorded as the predicted delta. The deltas of the measures clinically evaluated at time T1vsT0 and predicted by the ClinCheck^®^ at T1CvsT0 were statistically different for all the variables examined (Table 1).

**Figure 3:**
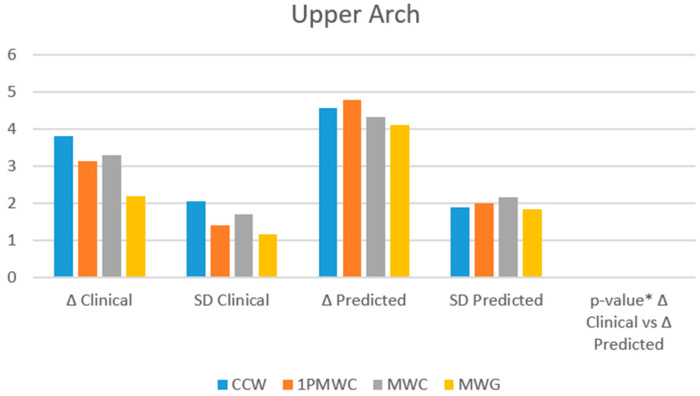
Clinical and predicted expansion values for the maxillary arch across key indicators. * paired *t* test.

The percentage of predictability was also calculated, showing acceptable values and therefore a moderate reliability of the ClinCheck^®^ software (Figure 3).

Table 2 shows the mean and SD of the measurements performed at T1 and T1C and shows the difference between these values.

The intraclass correlation coefficient evaluates reliability, as it considers both the degree of correlation and the agreement between measurements [18].

Reliability was moderate to excellent for 1PMWG (CIC = 0.89, 95% CI 0.56–0.97) and MWG (ICC = 0.90, 95% CI 0.59–0.97). For the other measures, reliability was good, although there was great variability, as indicated by the width of the 95% confidence intervals. Reliability was only not statistically significant for CGW (Table 3).

### 3.2. Lower Arch

Pre- and post-treatment clinical measurements were compared, and the difference between T0 and T1 is reported under the clinical delta column. On all measured levels, a satisfactory expansion rate was achieved (Table 4).

Pre-treatment measurements were then compared to the ClinCheck^®^ predicted expansion value, and the difference was recorded as the predicted delta. The deltas of the measures clinically evaluated at T1vsT0 and predicted by the ClinCheck^®^ T1CvsT0 were statistically different for all the variables examined.

**Figure 4:**
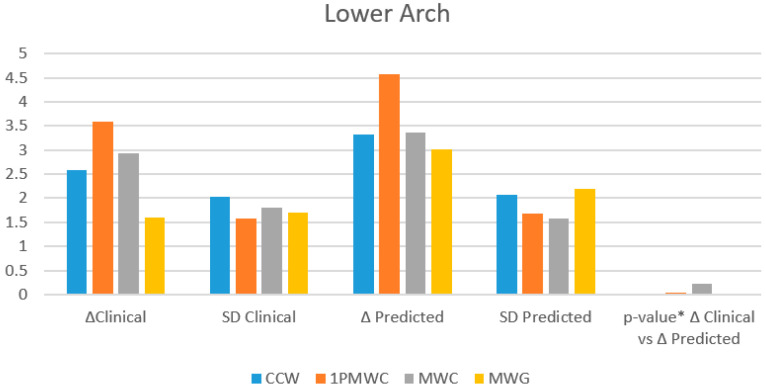
Clinical and predicted expansion values for the mandibulary arch across key indicators. * paired *t* test.

The percentage of predictability was also calculated, demonstrating summarily high values and, therefore, a good reliability of the ClinCheck^®^ software even in the mandibular arch (Figure 4).

Table 5 shows the mean and SD of the measurements performed at T1 and T1C and shows the difference between these values.

Reliability was good to excellent for MWC (ICC = 0.95, 95% CI 0.81–0.99, *p* < 0.001) and moderate to excellent for CCW (ICC = 0.87, 95% CI 0.48–0.97, *p* = 0.003), 1PMWG (ICC = 0.91, 95% CI 0.65–0.98, *p* = 0.001), and MWG (ICC = 0.92, 95% CI 0, 66–0.98, *p* = 0.001). For 2PMWG, the reliability was good, but there was wide variability (ICC = 0.86, 95% CI 0.44–0.97, *p* = 0.004). For CGW, 1PMWC, and 2PMWC, the reliability was not statistically significant (Table 6).

Complete results for all evaluated indicators are available in Supplementary Tables S1–S6.

## 4. Discussion

Previous studies have analyzed maxillary and mandibular arch expansion in growing patients using traditional appliances. For example, the study by Perillo et al. [19] evaluated the effects of a mixed palatal expansion protocol using a Hyrax expander, reporting significant increases in upper and lower arch widths. While their protocol focused primarily on skeletal expansion, particularly in the maxilla, our findings suggest that Invisalign First^®^ can also achieve clinically meaningful dentoalveolar expansion, even though it relies on different biomechanics (buccal tipping and controlled bodily movement). Despite the differences in treatment modality and mechanism of action, both approaches appear effective in increasing arch dimensions in prepubertal patients.

Although knowledge related to the Invisalign^®^ treatment methodology has been significantly expanded in recent years by developing numerous studies on the effectiveness of transparent aligners in the treatment of the maxillary arch, both in adults and in growing patients, in addition to clinical effectiveness, the mechanical behavior of aligner materials plays a key role in treatment success, particularly in long-term and growing patients. Recent studies have investigated how 3D-printed aligner materials respond to aging and prolonged intraoral exposure. Paradowska-Stolarz et al. [20] demonstrated that aligners exhibit significant variability in stress relaxation, force decay, and structural stiffness depending on the polymer composition and manufacturing process. Similarly, Paradowska-Stolarz et al. [21] showed that the aging process under intraoral-like conditions can reduce the mechanical integrity of aligners, affecting force delivery over time. These findings underline the need for careful material selection in pediatric orthodontics, where treatment duration may extend over months and involve biologically dynamic conditions. Invisalign^®^ aligners are manufactured using a proprietary SmartTrack^®^ material, which has been shown to provide better elasticity, consistent force application, and superior performance in long-term wear compared to some alternative resins used in other systems. This material advantage may help improve control of tooth movement and enhance predictability, particularly for expansion protocols that require sustained, light forces in growing patients.

Evidence is still lacking scientific studies on the predictability of mandibular expansion with Invisalign First^®^ in mixed dentition, so this study aimed to evaluate the effects in terms of alveolar expansion, arch width, arch perimeter, and molar inclination in both the dental arches in a sample of patients with mixed dentition using the Invisalign First^®^ system. Furthermore, another aim of this investigation was to evaluate the predictability of these movements by comparing actual results collected from clinical measurements with virtual ones expected from ClinCheck^®^.

Other studies have been conducted on a sample of patients with mixed dentition to evaluate the effectiveness of transparent aligners in maxillary expansion, but none of them focused on both arches and the predictability of ClinCheck^®^. Recent evidence has also highlighted variations in predictability between virtual plans and clinical outcomes in both growing and adult patients [22,23].

The present study is the first one in the scientific literature that focused its attention on the lower arch in patients with mixed dentition and added the intraclass correlation coefficient to the simple comparison between the pre- and post-treatment results to verify the actual reliability of ClinCheck^®^.

Although this is the first study to systematically evaluate mandibular arch expansion using clear aligners in mixed dentition, previous clinical evidence suggests that clear aligners may influence mandibular positioning. For example, mandibular arch changes were also observed in the retrospective study by Rocha et al. [24], although their analysis primarily focused on maxillary coronal expansion and reported mandibular changes only as secondary effects, without dedicated statistical evaluation. Additionally, a case series by Staderini et al. [25], including Meuli, documented favorable mandibular changes during anterior crossbite correction in growing patients, providing an early clinical foundation for aligner-driven modifications in the lower arch.

The results of the present study showed that adequate dentoalveolar expansion can be achieved with clear aligners in both the lower and upper arch, even in growing patients. An increase in transverse dimension occurred at all levels under measurement, with the greatest expansion being in the maxillary arch at the level of the second premolar (4.16 ± 2.48), followed by canines, molars, and first premolars. In the mandibular arch, the greatest dento-alveolar expansion was detected at the level of the second premolar (3.64 ± 1.38) and the first premolar (3.59 ± 1.57).

Furthermore, the tooth movements obtained met the expectations of ClinCheck^®^ with an average good predictability. In the upper arch, the highest percentage of predictability was found at the cusp level of the canine (84%), followed by molars (76%), first primary molar (75%), and second primary molar (66%). At all levels, gingival were shown to have lower predictability, showing a tendency towards tipping movement rather than bodily translation. In the lower arch, the highest value was recorded at the cusp level of the molars (88%), followed by the canines and first primary molar (78%) and second primary molar (65%). At the gingival level, predictability was lower but still good.

The greatest disagreement between cuspal and gingival percentages was found in canines in the maxillary arch and in molars in the mandibular arch, suggesting greater inclination of these teeth.

Only Lione et al. [26] studied the variations in the dimensions of the transverse maxillary arch using the Invisalign First System^®^ in subjects with early mixed dentition and observed the percentage of actual transverse expansion compared to that planned by ClinCheck^®^. Their results were in agreement with the present study, as the maximum predictability was at the level of the first deciduous molar (83%), followed by the deciduous canine (81%), the second deciduous molar (79%), and the first deciduous molar (77%), obtaining predictability results slightly higher than those presented in this study, except for the canine, which proved to be the most predictable, followed by the other teeth in the same order identified by Lione et al. [26]. Unfortunately, their study did not perform measurements at either the gingival or lower jaw level, so comparisons with these values cannot be made.

Other studies investigated dental arch expansion only in adult subjects, such as the 2020 study by Zhou and Guo [27], which studied the expansion efficiency of the upper arch using the Invisalign system on a sample of adult subjects; their results, similar to the present study, showed that the expansion efficiency at the cusp level of the first molar was 68.31 ± 24.41%, while the bodily expansion efficiency of the first molar was 36.35 ± 29.32%, and the average root-to-crown expansion movement ratio was 2:5.

Therefore, when a large amount of expansion is needed, clinicians should consider reducing the amount of expansion for each aligner to preserve periodontal health and preset a more negative coronal torque to better control crown and root movements, achieve bodily expansion, and avoid excessive buccal inclination of the posterior teeth and its negative effects on occlusion.

In 2021, Vidal-Bernárdez et al. [28], in a retrospective study conducted on a sample of 167 patients in permanent dentition undergoing expansion of the lower and upper arch, stated that the overall predictability for the upper arch was 92.49%, 99.15% at the canine level, and 85.84% at the gingival level. Instead, the average predictability of the lower arch was 88%, 98.9% at the canine level, and 76.4% at the gingival level. These results show greater predictability in the maxillary arch and less predictability at the gingival level in both the mandibular and maxillary arches due to the inclination movement of the crown.

In 2017 Houle et al. [29] and Solano-Mendoza et al. [10] studied a comparison between ClinCheck^®^ and 3D digital models, drawing the same conclusions, but they focused only on permanent dentition.

Recent evidence has also highlighted variations in predictability between virtual plans and clinical outcomes in both growing and adult patients [22,23].

From the results obtained in this study, however, it can be stated that good levels of dento-alveolar expansion can be achieved in the lower arch by considering the typical growth characteristics of the mandible during treatment planning. In fact, while the software proposes a simultaneous expansion of the arch based on the size of the crowns present in the arch, the clinician must predict the mutations that will occur during the exchange phase and calculate the E space that will be gained following the exfoliation of the second deciduous molars, thus avoiding excessive buccal inclination of the dental elements. Therefore, an initial slight crowding at the end of treatment in the presence of the second deciduous molar in the arch must be considered physiological, since following the exchange, the condition will undergo spontaneous correction. In the mandibular arch, therefore, ClinCheck^®^ is programmed to confer the correct amount of force to the dental elements, but a slight deviation from the pre-visualization of the expected result with a consequent slightly reduced predictability becomes completely normal and indeed desirable.

On the other hand, a 22–35% overengineering of the buccal movements could be planned by the clinician to achieve the desired amount of expansion, as an overengineering of the buccal root torque could allow for reducing the gap between the cusp and gingival points expansion observed in the present study, and this could allow for the achievement of a more bodily movement. Anyway, a bodily movement in mixed dentition could be considered important only on permanent molars, as the deciduous teeth will be subjected to exfoliation.

The present study has several limitations. First, the small sample size may reduce the statistical power and limit the generalizability of the findings.

In addition, no analysis was performed to assess the relationship between the planned expansion and the degree of predictability achieved.

The absence of a control group treated with conventional expansion methods also limits the ability to compare the effectiveness of clear aligners with traditional approaches. Furthermore, no follow-up evaluation was conducted, preventing assessment of long-term stability or potential relapse of dentoalveolar changes. Another limitation is the lack of skeletal maturity assessment (e.g., CVM stage), which may influence the biological response to expansion forces in growing patients. Patient compliance and inter-operator variability were not recorded, and factors such as oral hygiene or periodontal status were not systematically monitored, all of which could impact treatment outcomes. While measurement reliability was addressed through ICC analysis, further prospective studies are needed to confirm these findings and explore these variables more comprehensively.

In addition, two recent studies have further explored the predictability of transverse changes with clear aligners and Invisalign^®^ First, supporting the need for ongoing evaluation and refinement of virtual treatment planning [22,23].

It would be useful to perform future studies to improve these aspects.

## 5. Conclusions

Clear aligners can be considered an efficient device for the treatment of maxillary and mandibular dento-alveolar expansion in patients with mixed dentition.

ClinCheck^®^ has good predictability in both the upper and lower arch, more so at the coronal rather than gingival level, due to the tipping movement that the dental elements undergo.

Even if the aligner does not express the total planned amount of expansion (65–88%), overengineering of the buccal movement could be useful in allowing the clinician to achieve the desired amount of expansion with additional aligner orders.

## Figures and Tables

**Table 1:** Clinical and virtual measurements in the upper arch.

Upper Arch	Clinical Measuresments T0	Clinical Measurements T1	ΔClinical	Predicted Change T1	ΔPredicted	% of Predictability	*p*-Value * ΔClinical vs. ΔPredicted
CCW	31.83 ± 2.27	35.65 ± 1.04	3.81 ± 2.05	36.39 ± 0.78	4.56 ± 1.89	84%	0.012
1PMWC	39.68 ± 1.73	42.83 ± 1.71	3.15 ± 1.40	44.48 ± 1.64	4.80 ± 2.00	66%	0.005
MWC	50.58 ± 1.86	53.89 ± 1.11	3.31 ± 1.70	54.91 ± 1.46	4.33 ± 2.16	76%	0.017
MWG	32.89 ± 1.31	35.07 ± 1.99	2.18 ± 1.17	36.99 ± 2.41	4.10 ± 1.84	53%	0.001

Data are shown as mean ± standard deviation; * paired *t* test.

**Table 2:** Concordance data between clinical measurements and predicted changes in T1.

Variables	Clinical Measurement T1	Predicted Change T1	Difference Between Clinical and Predicted Measurements at T1	Mean (Clinical + Predicted)/2
CCW	35.65 ± 1.04	36.39 ± 0.78	−0.74	36.02
1PMWC	42.83 ± 1.71	44.48 ± 1.64	−1.65	43.65
MWC	53.89 ± 1.11	54.91 ± 1.46	−1.02	54.40
MWG	35.07 ± 1.99	36.99 ± 2.41	−1.92	36.03

**Table 3:** Intraclass correlation coefficients (ICCs) of measurements using clinical and virtual measurements (ModT-CkT).

	ICC	95% Interval Confidence		F Test with True Value 0	
		Lower	Upper	Value	*p* Value
CCWMod-CCWCC				4.89	0.013
Single measures (intraclass)	0.66	0.10	0.90		
Avarege measures (interclass)	0.80	0.18	0.95		
1PMWCMod-1PMWCCC				4.51	0.018
Single Measures (intraclass)	0.64	0.06	0.90		
Avarege Measures (interclass)	0.78	0.11	0.94		
MWCMod-MWCCC				4.55	0.017
Single measures (intraclass)	0.64	0.06	0.90		
Avarege measures (interclass)	0.78	0.12	0.95		
MWGMod-MWGCC				9.87	0.001
Single measures (intraclass)	0.82	0.42	0.95		
Avarege measures (interclass)	0.90	0.59	0.97		

**Table 4:** Clinical and virtual measurements in the lower arch.

Lower Arch	Clinical Measurements T0	Clinical Measurements T1	ΔClinical	Predicted Change	ΔPredicted	% of Predictability	*p*-Value * ΔClinical vs. ΔPredicted
CCW	25.17 ±1.63	27.76 ±1.08	2.59 ± 2.03	28.50 ± 1.03	3.33 ± 2.07	78%	0.009
1PMWC	31.70 ± 1.71	35.29 ± 1.17	3.59 ± 1.57	36.28 ± 1.14	4.58 ± 1.68	78%	0.037
MWC	44.43 ±1.56	47.36 ±2.31	2.94 ±1.80	47.78 ± 2.47	3.36 ± 1.57	88%	0.227
MWG	32.69 ± 1.73	34.29 ± 2.36	1.60 ± 1.70	35.70 ± 2.52	3.02 ± 2.20	53%	0.010

* paired *t* test.

**Table 5:** Concordance data between clinical measurements and expected changes at T1.

Variables	Clinical Measurement T1	Predicted Change T1	Difference Between Clinical and Predicted Measurements at T1	Mean (Clinical + Predicted)/2
CCW	27.76 ± 1.08	28.50 ± 1.03	−0.74	28.13
1PMWC	35.29 ± 1.17	36.28 ± 1.14	−0.99	35.78
MWC	47.36 ± 2.31	47.78 ± 2.47	−0.42	47.57
MWG	34.29 ± 2.36	35.70 ± 2.52	−1.42	34.99

**Table 6:** Intraclass correlation coefficients (ICC) of measurements using clinical and virtual measurements (ModT-CkT) (lower jaw).

	ICC	95% Interval Confidence		F Test with True Value 0	
		Lower	Upper	Value	*p* Value
CCWMod-CCWCC				7.73	0.003
Single measures (intraclass)	0.77	0.32	0.94		
Avarege measures (interclass)	0.87	0.48	0.97		
1PMWCMod-1PMWCCC				2.21	0.126
Single measures (intraclass)	0.38	−0.29	0.80		
Avarege measures (interclass)	0.55	−0.82	0.89		
MWCMod-MWCCC				20.83	<0.001
Single measures (intraclass)	0.91	0.68	0.98		
Avarege measures (interclass)	0.95	0.81	0.99		
MWGMod-MWGCC				11.78	0.001
Single measures (intraclass)	0.84	0.49	0.96		
Avarege measures (interclass)	0.92	0.66	0.98		

## Data Availability

The datasets used and/or analyzed during the current study are available from the corresponding author on reasonable request.

## Supplementary Materials

The following supporting information can be downloaded at: https://www.mdpi.com/article/10.3390/healthcare13131508/s1, Supplementary Tables S1–S6 are available as a separate file and report the full dataset of clinical and predicted measurements, statistical comparisons, and reliability values.
