# Supplementary material for: Efficacy and Predictability of Maxillary and Mandibular Dental Arch Expansion with Clear Aligners in Prepuberal Subjects: A Digital Retrospective Analysis
Source: Healthcare (Basel). 2025 Jun 24;13(13):1508. doi: 10.3390/healthcare13131508 (PMC12249088; doi:10.3390/healthcare13131508)
Supplement: Supplementary file 1 [file healthcare-13-01508-s001.zip › Table S1.pdf]

Table S1. Clinical and virtual measurements in the upper arch

Upper arch	Clinical measurements T0	Clinical measurements T1	Δ Clinical	Predicted Change T1	Δ Predicted	% of Predictability	p-value*	Δ Clinical vs Δ Predicted
CCW	31.83 ±2.27	35.65 ±1.04	3.81 ±2.05	36.39 ±0.78	4.56 ±1.89	84%	0.012	
CGW	25.06 ±1.50	26.75 ±1.58	1.69 ±0.59	29.53 ±1.35	4.47 ±1.93	38%	0.001	
1PMWC	39.68 ±1.73	42.83 ±1.71	3.15 ±1.40	44.48 ±1.64	4.80 ±2.00	66%	0.005	
1PMWG	27.86 ±1.41	30.37 ±2.13	2.52 ±1.28	32.54 ±2.50	4.68 ±1.92	54%	0.001	
2PMWC	44.74 ±1.52	48.91 ±2.11	4.16 ±2.48	50.28 ±1.89	5.54 ±2.18	75%	0.019	
2PMWG	30.38 ±1.85	33.11 ±2.09	2.73 ±1.28	35.61 ±2.72	5.23 ±2.22	52%	0.001	
MWC	50.58 ±1.86	53.89 ±1.11	3.31 ±1.70	54.91 ±1.46	4.33 ±2.16	76%	0.017	
MWG	32.89 ±1.31	35.07 ±1.99	2.18 ±1.17	36.99 ±2.41	4.10 ±1.84	53%	0.001	
	±1.31	±1.99	±1.17	±2.41	±1.84			
