# Supplementary material for: Efficacy and Predictability of Maxillary and Mandibular Dental Arch Expansion with Clear Aligners in Prepuberal Subjects: A Digital Retrospective Analysis
Source: Healthcare (Basel). 2025 Jun 24;13(13):1508. doi: 10.3390/healthcare13131508 (PMC12249088; doi:10.3390/healthcare13131508)
Supplement: Supplementary file 1 [file healthcare-13-01508-s001.zip › Table S2.pdf]

Table S2. Concordance data between clinical measurements and predicted changes in T1

Variables	Clinical measurement T1	Predicted Change T1	Difference between Clinical and Predicted Measurements at T1	Mean (clinical+predicted)/2
CCW	35.65±1.04	36.39±0.78	-0.74	36.02
CGW	26.75±1.58	29.53±1.35	-2.78	28.14
1PMWC	42.83±1.71	44.48±1.64	-1.65	43.65
1PMWG	30.37±2.13	32.54±2.50	-2.17	31.46
2PMWC	48.91±2.11	50.28±1.89	-1.37	49.59
2PMWG	33.11±2.09	35.61±2.72	-2.50	34.36
MWC	53.89±1.11	54.91±1.46	-1.02	54.40
MWG	35.07±1.99	36.99±2.41	-1.92	36.03
