# Supplementary material for: Efficacy and Predictability of Maxillary and Mandibular Dental Arch Expansion with Clear Aligners in Prepuberal Subjects: A Digital Retrospective Analysis
Source: Healthcare (Basel). 2025 Jun 24;13(13):1508. doi: 10.3390/healthcare13131508 (PMC12249088; doi:10.3390/healthcare13131508)
Supplement: Supplementary file 1 [file healthcare-13-01508-s001.zip › Table S3.pdf]

Table S3. Intraclass correlation coefficients (ICCs) of measurements using clinical and virtual measurements (ModT-CkT)

Variables	ICC	95% Confidence Interval		F test with True value 0	p value
		Lower	Upper		
CCWMod - CCWCC	0.66	0.10	0.90	4.89	0.013
	0.80	0.18	0.95		
CGWMod - CGWCC	0.20	-0.45	0.72	1.51	0.274
	0.34	-1.66	0.84		
1PMWCMod - 1PMWCCC	0.64	0.06	0.90	4.51	0.018
	0.78	0.11	0.94		
1PMWGMod - 1PMVGCC	0.80	0.39	0.95	9.25	0.001
	0.89	0.56	0.97		
2PMWCMod - 2PMWCCC	0.71	0.20	0.92	6.01	0.007
	0.83	0.33	0.96		
2PMWGMod - 2PMWGCC	0.75	0.28	0.93	7.11	0.004
	0.86	0.43	0.97		
MWCMod - MWCCC	0.64	0.06	0.90	4.55	0.017
	0.78	0.12	0.95		
MWGMod - MWGCC	0.82	0.42	0.95	9.87	0.001
	0.90	0.59	0.97		
