# Supplementary material for: Efficacy and Predictability of Maxillary and Mandibular Dental Arch Expansion with Clear Aligners in Prepuberal Subjects: A Digital Retrospective Analysis
Source: Healthcare (Basel). 2025 Jun 24;13(13):1508. doi: 10.3390/healthcare13131508 (PMC12249088; doi:10.3390/healthcare13131508)
Supplement: Supplementary file 1 [file healthcare-13-01508-s001.zip › Table S4.pdf]

Table S4. Clinical and virtual measurements in the lower arch

Lower arch	Clinical measurements T0	Clinical measurements T1	ΔClinical	Predicted Change	Δ Predicted	% of predictability	p-value *
CCW	25.17 ±1.63	27.76 ±1.08	2.59 ±2.03	28.50 ±1.03	3.33 ±2.07	78%	0.009
CGW	20.14 ±1.17	10.43pm ±1.34	2.30 ±0.98	11.58pm ±1.03	3.44 ±1.29	67%	0.026
1PMWC	31.70 ±1.71	35.29 ±1.17	3.59 ±1.57	36.28 ±1.14	4.58 ±1.68	78%	0.037
1PMWG	24.24 ±1.79	27.84 ±2.08	3.59 ±1.83	29.48 ±2.29	5.24 ±1.84	69%	0.002
2PMWC	37.14 ±1.10	40.78 ±1.45	3.64 ±1.38	42.73 ±1.56	5.59 ±1.88	65%	0.010
2PMWG	27.85 ±1.62	30.76 ±2.58	2.91 ±1.48	33.50 ±2.76	5.65 ±2.17	52%	0.001
MWC	44.43 ±1.56	47.36 ±2.31	2.94 ±1.80	47.78 ±2.47	3.36 ±1.57	88%	0.227
MWG	32.69 ±1.73	34.29 ±2.36	1.60 ±1.70	35.70 ±2.52	3.02 ±2.20	53%	0.010
