# Supplementary material for: Efficacy and Predictability of Maxillary and Mandibular Dental Arch Expansion with Clear Aligners in Prepuberal Subjects: A Digital Retrospective Analysis
Source: Healthcare (Basel). 2025 Jun 24;13(13):1508. doi: 10.3390/healthcare13131508 (PMC12249088; doi:10.3390/healthcare13131508)
Supplement: Supplementary file 1 [file healthcare-13-01508-s001.zip › Table S5.pdf]

Table S5. Concordance data between clinical measurements and expected changes at T1

Variables	Clinical measurement T1	Predicted Change T1	Difference between Clinical and Predicted Measurements at T1	Mean (clinical+predicted)/2
CCW	27.76±1.08	28.50±1.03	-0.74	28.13
CGW	22.43±1.34	23.58±1.03	-1.14	23.00
1PMWC	35.29±1.17	36.28±1.14	-0.99	35.78
1PMWG	27.84±2.08	29.48±2.29	-1.65	28.66
2PMWC	40.78±1.45	42.73±1.56	-1.95	41.75
2PMWG	30.76±2.58	33.50±2.76	-2.74	32.13
MWC	47.36±2.31	47.78±2.47	-0.42	47.57
MWG	34.29±2.36	35.70±2.52	-1.42	34.99
