# Supplementary material for: Efficacy and Predictability of Maxillary and Mandibular Dental Arch Expansion with Clear Aligners in Prepuberal Subjects: A Digital Retrospective Analysis
Source: Healthcare (Basel). 2025 Jun 24;13(13):1508. doi: 10.3390/healthcare13131508 (PMC12249088; doi:10.3390/healthcare13131508)
Supplement: Supplementary file 1 [file healthcare-13-01508-s001.zip › Table S6.pdf]

Table S6. Intraclass correlation coefficients (ICC) of measurements using clinical and virtual measurements (ModT-CkT) (lower jaw)

Variables	ICC	95% Confidence Interval		F test with True value 0	p value
		Lower	Upper		
CCWMod - CCWCC	0.77	0.32	0.94	7.73	0.003
	0.87	0.48	0.97		
CGWMod - CGWCC	0.36	-0.31	0.79	2.11	0.141
	0.53	-0.91	0.88		
1PMWCMod - 1PMWCCC	0.38	-0.29	0.80	2.21	0.126
	0.55	-0.82	0.89		
1PMWGMod - 1PMVGCC	0.84	0.48	0.96	11.43	0.001
	0.91	0.65	0.98		
2PMWCMod - 2PMWCCC	0.19	-0.46	0.71	1.48	0.286
	0.32	-1.73	0.83		
2PMWGMod - 2PMWGCC	0.75	0.28	0.93	7.15	0.004
	0.86	0.44	0.97		
MWCMod - MWCCC	0.91	0.68	0.98	20.83	<0.001
	0.95	0.81	0.99		
MWGMod - MWGCC	0.84	0.49	0.96	11.78	0.001
	0.92	0.66	0.98		
